# High throughput automated microbial bioreactor system used for clone selection and rapid scale‐down process optimization

**DOI:** 10.1002/btpr.2534

**Published:** 2017-08-10

**Authors:** M. Lourdes Velez‐Suberbie, John P. J. Betts, Kelly L. Walker, Colin Robinson, Barney Zoro, Eli Keshavarz‐Moore

**Affiliations:** ^1^ The Advanced Centre for Biochemical Engineering Department of Biochemical Engineering, University College London Gower Street, Bernard Katz Building, London WC1E 6BT U.K.; ^2^ Sartorius Stedim Biotech York Way, Royston Herts SG8 5WY U.K.; ^3^ Centre for Molecular Processing School of Biosciences, University of Kent Canterbury CT2 7NJ U.K.

**Keywords:** *E. coli*, single use microbioreactor, high throughput, scale‐down, ambr 15 fermentation

## Abstract

High throughput automated fermentation systems have become a useful tool in early bioprocess development. In this study, we investigated a 24 x 15 mL single use microbioreactor system, ambr 15f, designed for microbial culture. We compared the fed‐batch growth and production capabilities of this system for two Escherichia coli strains, BL21 (DE3) and MC4100, and two industrially relevant molecules, hGH and scFv. In addition, different carbon sources were tested using bolus, linear or exponential feeding strategies, showing the capacity of the ambr 15f system to handle automated feeding. We used power per unit volume (P/V) as a scale criterion to compare the ambr 15f with 1 L stirred bioreactors which were previously scaled‐up to 20 L with a different biological system, thus showing a potential 1,300 fold scale comparability in terms of both growth and product yield. By exposing the cells grown in the ambr 15f system to a level of shear expected in an industrial centrifuge, we determined that the cells are as robust as those from a bench scale bioreactor. These results provide evidence that the ambr 15f system is an efficient high throughput microbial system that can be used for strain and molecule selection as well as rapid scale‐up. © 2017 The Authors Biotechnology Progress published by Wiley Periodicals, Inc. on behalf of American Institute of Chemical Engineers *Biotechnol. Prog.*, 34:58–68, 2018

## Introduction

Great effort has been put into biopharma process development specifically strain or media screening and fermentation optimization to increase product yield and reduce product development timelines.^1^, ^2^, ^3^, ^4^ While shake flask systems have been widely used for strain/product screening, they have the disadvantage in that they lack automated feeding, pH and oxygen control^5^, ^6^ which potentially create an undesirable environment for cell growth. As a result, parallel small/mini bioreactors have become a more desirable method in early stages of process development as they have the capability to control and mimic the conditions that the cells will experience in a larger vessel.^5^, ^7^, ^8^, ^9^ This has led to the development of automated high throughput fermentation systems as they provide better early stage process understanding.^1^, ^2^, ^10^ There are many high throughput systems commercially available for fermentation of mammalian and microbial cells, including Applikon's micro‐matrix (24 × 1 to 5 mL, microbial and mammalian), the micro24 from Pall^11^, ^12^, ^13^, ^14^, ^15^ (24 × 3 to 7 mL, aerobic and anaerobic microbial fermentation, mammalian and insect cell culture), Biolector^®^ from m2p labs^16^, ^17^ (32 × 800 µL to 2,000 µL mammalian and microbial), 2mag bioreactor from 2mag AG (8 or 48 × 8 to 15 mL, aerobic and anaerobic microorganism)^18^ and ambr^®^ 15 cell culture^5^, ^15^, ^19^, ^20^, ^21^ (24 × 10 to 15 mL, mammalian).

ambr 15 cell culture has been commonly used and tested showing comparable cell culture performance and productivity to different scale bench top bioreactors i.e., 2 L,^22^ 3 L,^19^ 5 L.^5^, ^20^, ^23^ It has also been shown that the ambr 15 can mimic pilot plant scale (15 and 200 L^23^) and manufacturing scale (up to 15,000 L^20^) bioreactor systems. The recent development of the microbial ambr 15 fermentation system (ambr 15f) has overcome the limitations of other microbial microbioreactor systems as it has the capability of individual monitoring and control of dissolved oxygen and pH. The inclusion of pumped liquid addition lines allows for feed and base to be pumped to individual vessels as required, and pipetting operations can be automated to trigger induction after a culture event, for example. The ambr 15f, as previously demonstrated in the ambr 15 cell culture,^19^, ^20^ has the capability to be used for design of experiments (DoE); the hardware and software easily lends itself to this manner of experimentation. Desired parameters can be DoE tagged in the ambr 15f software and the range of process set points imported from a DoE software package. Data from the ambr 15f, experiment can then be exported from the ambr 15f software into a DoE software package for analysis.

In this study, we have addressed two issues. First, we have evaluated the bioreactor reproducibility both within and across culture stations (blocks of 12 bioreactor vessels) of the ambr^®^ 15 fermentation, in fed‐batch mode for microbial cell growth and production of heterologous proteins. Second, we have demonstrated that this automated high throughput microbioreactor system can be used as scale‐down tool for microbial fermentation.

## Materials and Methods

Chemicals, unless specified otherwise, were obtained from Sigma Chemical Co. Ltd. (Poole, Dorset, UK).

### Bacterial strains and molecules

*E. coli* MC4100 cells expressing two single chain variable fragment antibodies (scFv). scFv raised against omega peptide of β‐galactosidase, designated as scFv_1_ (courtesy of Cobra Biologics),^24^ scFv anti‐c‐Met, designated as scFv_2_ (courtesy of James Austerberry, University of Manchester).^25^ BL21 (DE3) cells expressing an scFv IL1B, designated as scFv_3_ (kind donation of Prof Lloyd Ruddock, University of Oulu) and human growth hormone (hGH).^24^ scFv_1_, scFv_2_ and hGH were translocated to the periplasm (P).

### Seed culture preparation

Seed cultures were inoculated with 15 μL of glycerol stocks and cells were grown overnight in 5 mL of Luria broth (LB) in an orbital shaker incubator at 30°C, 250 rpm. Overnight cultures were used to inoculate flasks with 200 mL of LB supplemented with antibiotics (100 μg mL^−1^ of ampicillin or 50 μg mL^−1^ of kanamycin) and were grown for 3 h (30°C, 250 rpm). These were used as a 10% (v/v) inoculum for 200 mL of defined medium^26^ supplemented with the corresponding antibiotics and cells were incubated to an OD_600 nm_ of 5 at 30°C, 250 rpm for about 10 h.

### Fed‐batch fermentations

Fed‐batch fermentations were performed using an ambr^®^ 15 fermentation (ambr 15f) system (Sartorius Stedim Biotech, Royston, UK) initial working volume 10 mL (maximum volume 15 mL). The ambr 15f has two culture stations (CS1 and CS2), each one containing 12 single use microbioreactors. The system has a liquid handler for reactor set up and automated pipetting operations. Each culture station has independent stirring control and controlled background air flow with individual vessel heaters for fine temperature control. The system has pumped liquid lines to all individual vessels for feed and base additions delivering 5 μL shots. The vessel includes a single Rushton‐like impeller, sub‐surface sparge, feed lines, a line for liquid surface delivery of base and optical sensors for pH and DO monitoring (12 s cycle time).

Defined medium was supplemented to 90 g L^−1^ of glycerol in all fermentations, except for the fermentations where two carbon sources were used, see details in materials and methods section glucose and glycerol fermentations. Microbioreactors were supplemented with polypropylene glycol 2000 (PPG) to a final concentration of 0.01% (v/v) and antibiotics (100 μg mL^−1^ of ampicillin or 50 μg mL^−1^ of kanamycin) prior to inoculation. The microbioreactors were inoculated to an OD_600 nm_ of 0.3, with cells that have been adapted to grow on a defined medium. pH was measured on‐line using fluorescent sensor patches and the pH was held at 6.95 using 15% (v/v) ammonia solution or 15% (v/v) H_2_SO_4_. Base was added using the pumped liquid delivery system and acid using the liquid handler; the lower limit was controlled at 6.90 and the upper limit at 7.20. The upper pH limit control was activated after the first pH spike which was used to indicate the end of the batch phase. The pH was monitored off‐line using the analysis module (AM) which provides an automated, in‐process pH sensor re‐calibration as necessary. Dissolved oxygen tension (DOT) was measured on‐line using fluorescent sensor patches and was maintained at 30% using 1 vvm of air or air/oxygen as required, temperature was maintained at 30°C ± 0.20. To prevent foaming, 100 μL bolus additions of a 10% (v/v) PPG 2000 solution were performed at regular intervals from inoculation to induction using the liquid handler. The microbioreactors were supplemented with 15 mM magnesium sulfate (2.0 M stock) and 35 mM sodium phosphate (2.0 M stock, pH 6.5 at 25°C) when the cells grew to an OD_600 nm_ of 30–40. Feeding and induction were triggered on an individual vessel basis by way of a control loop observing an increase in pH when the main carbon source was exhausted^26^, ^27^, ^28^, ^29^; cells were fed with 40% (w/w) glycerol at 3.2 mL L^−1^ h^−1^ using the pumped liquid delivery system and induced using the liquid handler in a single bolus addition to a final concentration in the vessels of 1 mM isopropyl‐beta‐D‐thiogalactopyranoside (IPTG). Cell growth was monitored off‐line by optical density measurements at 600 nm. Dry cell weight (DCW) of each vessel was measured in duplicate at harvest as per Branston et al.^30^

Fed‐batch fermentations were also performed in four 1 L Multifors fermenters (Infors UK Ltd., Reigate, UK). The seed cultures were prepared as for the ambr 15f system; the fermenters were inoculated to an OD_600 nm_ of 0.3. Fermentations were performed as per Matos et al.^31^ with the following modifications: working volume was 825 mL, pH was controlled at 6.95 ± 0.10 using 15% (v/v) ammonia solution or 15% (v/v) H_2_SO_4_ and the temperature was maintained at 30°C throughout the fermentation.

### Glucose and glycerol fermentations

**Figure 1:**
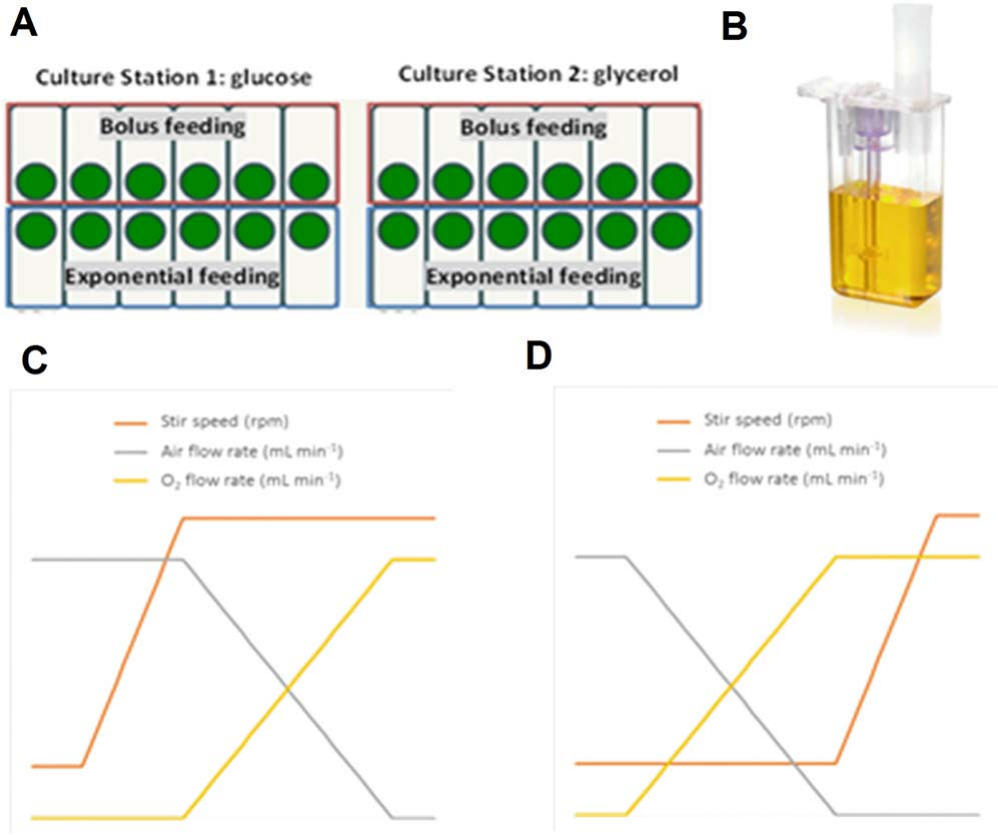
**A**: Schematic representation of the experimental layout in the ambr 15f system. **B**: ambr 15f vessel. **C** and **D**: Schematic representations of the control cascades strategies used for the scale‐down characterization: **C**: cascade A (air/stirrer speed/O_2_) and **D**: cascade B (air/O_2_/stirrer speed).

Two carbon sources and two feeding strategies were investigated in the ambr 15f system; Figure 1A shows the layout of the experimental design. The carbon sources were glucose (CS1) or glycerol (CS2) with an initial concentration of 30 g L^−1^ of either carbon source in the defined medium. The feeding strategy during the exponential phase were either bolus or continuous. When the carbon source was exhausted, as indicated by an increase in pH, the culture was supplemented with a 40% (w/w) glucose or glycerol solution to a final equivalent total concentration of 90 g L^−1^. For the bolus fed vessels, three additions of the 40% (w/w) solution were performed at regular intervals over 10 h. In the case of the continuously fed vessels, an exponential feeding method was used to control the theoretical maximum growth rate (*μ*_max_) at 0.30 h^−1^. Each feeding strategy was tested in half of the vessels of each culture station. At the end of exponential phase, a second pH spike was used to automatically activate constant feeding and induction; cells were fed with 40% (w/w) glycerol or glucose at 3.2 mL L^−1^ h^−1^ and induced at a final concentration of 1 mM (IPTG).

The starter cultures were prepared as mentioned in the materials and methods section, seed culture preparation. All other parameters of the fermentations were performed as described in the materials and methods section, fed‐batch fermentations.

### Scale‐down fermentations

Equal power per unit volume (P/V) was chosen as the scale down method.^32^, ^33^ The power number (N_P_) for the ambr 15f system was determined by the same method as described in Nienow et al.,^5^ N_P_ for Multifors was determined using the correlation with Reynolds number for Rushton turbine.^34^ For both systems, power (P) was calculated with: $\mathrm{P =}N_{P}\rho N_{i}^{3}D_{i}^{5}$ where N_P_ is the power number, ρ is fluid density, N_i_ is stirrer speed, D_i_ is impeller diameter.^5^ The P/V corresponding to the minimum and maximum stirrer speeds in the Multifors bioreactors were calculated (155 and 7,661 W m^−3^) and these values were used to determine the equivalent stirrer speed in the ambr 15f (Table 1). ambr 15f has a minimum stir speed of 500 rpm and maximum stir speed of 3,000 rpm. In addition, the volumetric mass transfer coefficient (k_L_a) at both scales was compared to ensure that this was not a limiting factor in the conditions tested. The k_L_a at both scales was comparable; 150 h^−1^ for the Multifors (determined by the dynamic gassing out method as described in Lamping et al.^35^) and between 150 and 180 h^−1^ for the ambr 15f system (estimated based on data included in the Supporting Information Table S1). To maintain the DO set point of 30% two cascade control strategies were investigated: (A) first increase stirrer speed and then the proportion of oxygen in the gas mix (i.e., air/stirrer speed/O_2_) (Figure 1C) or (B) increase oxygen percentage in gas mixture and then increase stirrer speed (i.e., air/O_2_/stirrer speed) (Figure 1D).

**Table 1:** Scale‐Down Parameters in the Multifors and ambr 15f System Using Constant P/V

	Impeller Characteristics				Stirrer Speed (min^−1^)	
	Type	Number	Diameter (mm)	Working Volume (mL)	Minimum	Maximum
Multifors	Rushton	2	38.0	825	300	1100
ambr 15f	Rushton‐like	1	11.4	10	710	2585

The seed cultures were prepared as described in the materials and methods section, fed‐batch fermentations. To reduce variability, the same inoculum was used for both systems (ambr 15f and Multifors) and the medium for the ambr 15f was autoclaved in the Multifors vessels, removed in a sterile manner and added to the ambr 15f vessels. The seeding of the fermenters and all other parameters of the fermentations were maintained/performed as described in materials and methods sections seed culture preparation and fed‐batch fermentations.

### Cell fractionation

Cells were fractionated into periplasm (P) and cytoplasm (C) by the EDTA/lysozyme/cold osmoshock method as described in Branston et al.^30^ Prior to fractionation the sample concentration was normalised to OD_600 nm_ of 10 per mL. All cell fractions were stored frozen in aliquots for further experiments and analysis.

### Cell lysis

The cells were lysed by freeze thawing as described in Gaciarz, et al.^36^ The cell concentration was normalised to an OD_600 nm_ of 10 per mL, cells were collected by centrifugation (15 min, 18,000 g) and resuspended in lysis buffer (50 mM sodium phosphate (pH 7.4), 20 μg mL^−1^ DNase and 0.1 mg mL^−1^ egg white lysozyme). Samples were incubated for 10 min and then frozen. The cell lysate was thaw and the soluble fraction was collected by centrifugation (15 min, 18,000 g).

### Protein purification

The His tag proteins were purified by immobilized metal affinity chromatography (IMAC) using HisPur Cobalt Superflow Agarose resin (Thermo Scientific, MA, USA). 5 mL of the soluble fraction were loaded in to the column and purified as described in Gaciarz, et al.^36^ The elution fraction was buffer exchanged into 50 mM sodium phosphate buffer (pH 7.4) using a Vivaspin 500 with a molecular weight cut off of 10,000 Da (GE Healthcare Life Sciences, Buckinghamshire, UK).

### Protein detection

The protein concentration from the cell fractions was determined by either western blot or high performance liquid chromatography (HPLC). For western blotting, reduced samples were prepared according to manufacturer's instructions and loaded onto a 12% NuPAGE Bis‐Tris gel electrophoresis (Invitrogen, Paisley, UK), then wet transferred using XCell II Blot Module to polyvinylidene difluoride membrane (PVDF) (Invitrogen) according to manufacturer's protocol. Membranes were immunoblotted with an anti‐His antibody (Invitrogen) the labeled bands were detected using enhanced chemiluminescence kit (BioRad, Herts, UK) according to the manufacturer's instructions. Membranes were scanned using a GE Typhoon scanner (GE Healthcare Life Sciences). The product concentration was determined by densitometry comparing against a known amount of purified material.

Protein L HPLC quantification was performed using an Agilent 1200 series HPLC system (Agilent Technologies, South Queensferry, UK) and 1 mL HiTrap^TM^ Protein L column (GE Healthcare). 100 μL of sample was loaded onto the column, the column was washed with 4 column volumes (CV) of 20 mM phosphate buffer (pH 7.2). Gradient elution with 20 mM phosphate buffer (pH 2.0) was used from a 0% to 100% concentration over 10 CV at 1 mL min^−1^. The column was re‐equilibrated with 3 CV of the wash buffer. A standard curve of purified scFv_1_ was used to determine the concentration of scFv_1_ in the fermentation samples.

IMAC HPLC quantification was performed using an Agilent 1200 series HPLC system and 1 mL HiTrap^TM^ IMAC HP column (GE Healthcare). The column was loaded with CoSO_4_ according to manufacturer's instructions. Hundred microliter of sample were loaded onto the column, the column was washed with 4 CV of 20 mM phosphate, 500 mM NaCl, 20 mM imidazole buffer (pH 7.4). Gradient elution with 20 mM phosphate, 500 mM NaCl, 500 mM imidazole buffer (pH 7.4) was used from a 0% to 100% concentration over 10 CV at 1 mL min^−1^. The column was re‐equilibrated with 3 CV of the wash buffer.

### Cell resistance to damage measurements

A rotating disk shear device was used to determine the relative resistance to damage of *E. coli* cells.^30^ At harvest, 20 mL of cell broth was exposed for 20 s to a rotation speed of 233 revolutions per second (rps) in the device. The rotational speed of 233 rps corresponds to an energy dissipation rate (EDR) of 0.75 × 10^6^ W kg^−1^,^37^ which is equivalent to the forces experienced in a continuous centrifuge.^30^ Pre and postshearing samples were centrifuged at 17,000*g* for 10 min. The supernatant was removed for analysis. The shearing was performed in triplicate for the 15 mL microbioreactors (cell broth from three microbioreactors was combined to satisfy the volume requirement of the shear device).

### Particle size distribution

The particle size distribution of pre and post sheared material was determined using Mastersizer 3000 (Malvern Instruments, Malvern, UK). A wet dispersion unit was used and each sample measurement was repeated five times. The size distribution of the sheared and nonsheared cells was analyzed using the nonspherical particle function within the Mastersizer software V3.10 (Malvern Instruments).

### Protein detection

The total soluble protein concentration from the pre and post sheared material was determined by Bradford assay (Thermo Scientific, IL, USA). The method was performed according to the manufacturer's instructions using 96 deep square well micro plates. Absorbance was measured at 595 nm using a plate spectrophotometer (Tecan Safire2, Tecan, Reading, UK).

### Statistical analysis

T test analysis or analysis of variance (ANOVA) were used for comparison of group mean. Both test were performed in OriginPro 9.1 (OriginLab Corporation, MA, USA).

## Results and Discussion

### Development of a robust process system

Parallel automated microbioreactor systems have the potential to shorten process/product development time lines by increasing the capacity to carry out many experiments simultaneously.^1^ To have a successful high throughput system, process robustness and system consistency are vital.^2^ ambr^®^ 15 fermentation system consistency was tested by measuring cell growth and productivity of an *E. coli* MC4100 cell line expressing scFv_1_ where all the microbioreactors were inoculated from the same pool of cells and the same set points and operating parameters were used in all vessels. At the end of exponential phase, the exhaustion of carbon source was indicated by DOT/pH spikes^26^, ^27^, ^28^, ^29^ pH spikes were used to trigger feed control loops, initiating pumped addition of a 40% (w/w) glycerol feed solution on an individual vessel basis. In addition the pH spikes also activated control loops which prompted the liquid handler to first add inducer (IPTG) and second take a sample from the specific vessels.

**Figure 2:**
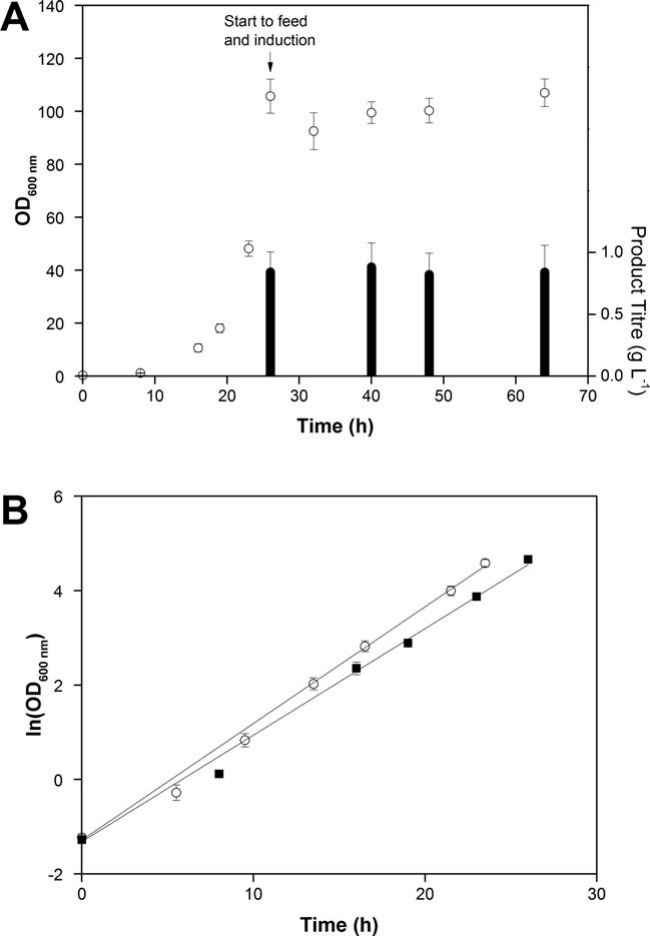
Process development and reproducibility tests in ambr 15f system. **A**: Growth curve during fedbatch fermentations of *E. coli* MC4100 cells grown in ambr 15f system. Cultures were induced with IPTG (1 mM) and fed with 40% (w/w) glycerol when carbon source was exhausted, *n* = 24 vessels Protein L HPLC results showing the scFv1 concentration in the periplasmic (P) fractions hours postinduction, *n* = 24 for 40 and 63 h; *n* = 6 for 32 and 48 h. **B**: Exponential growth phase of *E. coli* MC4100 cells from duplicate experiments 1 (▪) and 2 (○), *n* = 24 respectively.

Figure 2A shows the growth curves from *E. coli* MC4100 cells. The growth profile was consistent in the 24 vessels (12 vessels × 2 culture stations ran simultaneously), with a coefficient of variance between 3 and 7% over the 64 h of fermentation. The maximum OD 600 nm achieved was 100 and the DCW at the end of fermentation was 37.3 ± 2.93 g L^−1^. Affinity high performance liquid chromatography (Protein L) was used as a high throughput method to determine the concentration of the scFv_1_ in the periplasm (Figure 2A). The scFv_1_ concentration at harvest was 0.84 ± 0.22 g L^−1^; ANOVA test showed that the population variances at a level of 0.05 were not significant. The concentration of scFv_1_ in the extracellular medium was less than 0.1 g L^−1^ showing minimum product loss due to leakage into the extracellular medium. A second, repeat experiment indicated a consistent maximum growth rate between the two experiments (Figure 2B), with a *μ*_max_ of 0.25 ± 0.004 h^−1^ for experiment 1 and 0.23 ± 0.004 h^−1^ for experiment 2. ANOVA test (level of 0.05) showed no significant variances in the maximum growth rate, demonstraiting in‐run (intra run) consistency. The run‐run (inter run) consistency/reproducibilty falls within expected variability given that cultures were grown from two separate inocula.

In summary, we developed a process to grow *E. coli* cells in fed‐batch mode in the ambr 15f system and have showed that the cell growth and recombinant protein productivity within two culture stations and the 24 microbioreactors was reproducible.

**Figure 3:**
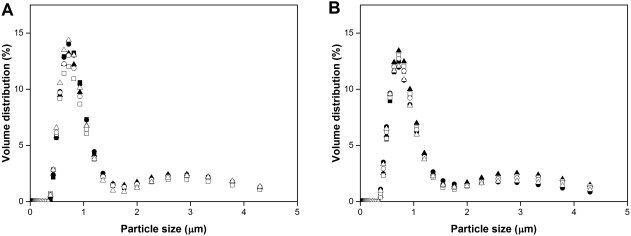
Volume distribution of *E. coli* MC4100 cells expressing scFv_1_ in fed‐batch fermentations grown in ambr 15f system. Samples from CS1 (•, ▪, filled triangles) and CS2 (○, □, △) **A**: pre exposure to high shear forces and **B**: post exposure to high shear forces.

Cell integrity has been shown to have an impact on downstream processing and product recovery^38^ for this reason ultra scale‐down (USD) technology was used to mimic the forces that the cells will experience during downstream processing. At the end of fermentation the cells grown in the ambr 15f system were exposed to a high energy dissipation rate (EDR) of 0.75 × 10^6^ W kg^−1^, in a rotating shear device^37^ where three vessels were pooled to satisfy the volume required for the device. No change was observed in the particle size distribution profile compared to the single vessels. The particle size distribution (Figure 3) and the soluble protein release assay (data not shown) indicated that the cells grown in the ambr 15f were able to withstand the shear forces present during downstream processes, especially in continuous centrifuges, this finding agreed with what had been previously shown by Branston et al.^30^ for *E. coli* cells cultivated in bench top bioreactors.

### Scaling characterization

The ambr 15f system was compared with 4 × 1 L Multifors bioreactors. The scale‐down criterion chosen to compare the nongeometrically similar systems was power per unit volume,^33^ the equivalent minimum and maximum stirrer speeds for P/V scale‐down fermentations are shown in Table 1. In addition, the *k*_L_ *a* of ambr 15f was compared with the Multifors to ensure that this was not a limiting factor. In the case of a limiting *k*_L_ *a* at small scale, the volumetric mass transfer coefficient could be improved by increasing the gas flow rate and/or reducing the working volume as demonstrated in Supporting Information Table S1 (Supporting Information Appendix).

**Figure 4:**
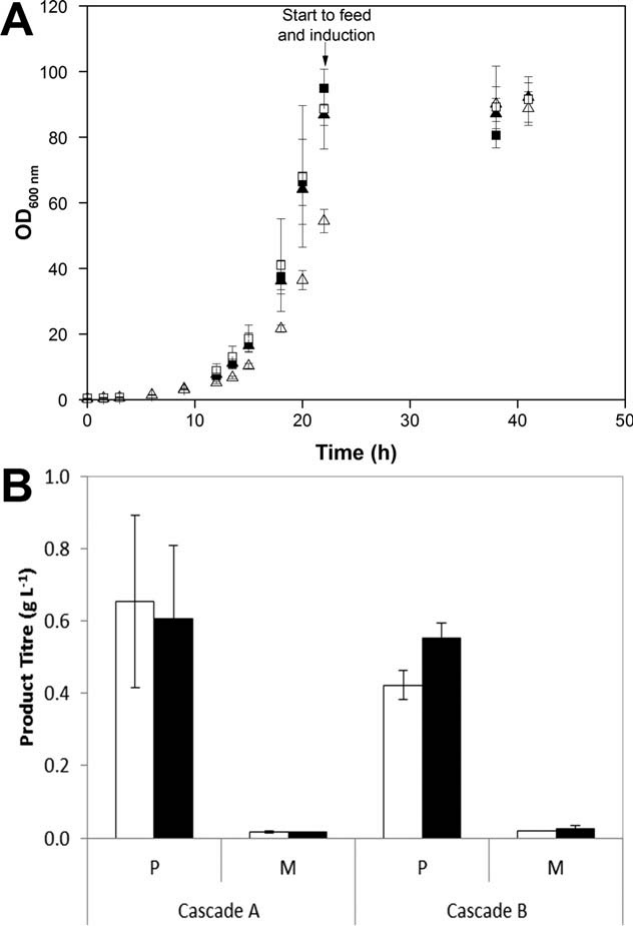
Scale‐down comparison of the ambr 15f and Multifors fermenters. **A**: Growth profile from fedbatch fermentations of *E. coli* MC4100 grown in parallel in ambr 15f (△, filled triangles) and Multifors bioreactors (□,▪), cascade control strategy A for filled symbols and B for the empty symbols. Number ofreplicates in Multifors = 2 and ambr 15f = 12. **B**: Protein L HPLC results showing the scFv_1_ concentration inthe medium (M) and periplasmic (P) fractions from the ambr 15f (□) and Multifors (▪) fermentations at 18 h postinduction.

**Figure 5:**
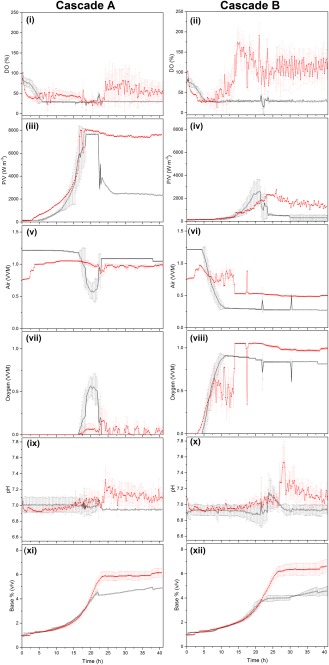
Fermentation traces for **i, ii**: dissolved oxygen (DO), **iii, iv**: power per unit volume (P/V), **v, vi**: air flow rate (VVM), **vii, viii**: oxygen flow rate (VVM), **ix, x**: pH and **xi, xii**: volume of base added (% (v/v)) for cascade A (air/stirrer speed/O_2_) and cascade B (air/O_2_/stirrer speed) and for ambr 15f (▪, red) and Multifors (□, black). For each cascade: *n* = 12 ambr 15f vessels and *n* = 2 Multifors vessels. N.B. No error bars shown for Multifors traces, cascade A after 20 h as *n* = 1.

The growth curves from the scale‐down experiments are shown in Figure 4A. The maximum OD in CS1 and the four 1 L fermenters was achieved 22 h after inoculation, with a matching *μ*_max_ of 0.27 ± 0.002 h^−1^, showing that the cells at both scales presented comparable growth rate. In the case of CS2 (cascade B, Figure 1D) the maximum growth rate was 0.23 ± 0.003 h^−1^, the cell growth in these vessels was lower than in CS1 (cascade A, Figure 1C) and in the Multifors fermenters (cascade A and B, Figures 1C, D), suggesting that the performance of the cells might have been affected by the cascade system used to control dissolved oxygen. ANOVA test showed that at a level of 0.05 the maximum growth rate from CS2 was significantly different from the one observed in CS1 and the 1 L fermenters. In the CS2 (cascade B), during the batch phase, the vessel with the faster growing cells drives the impeller speed (Figure 5 iv). As we have shown cell growth between vessels was highly consistent, therefore there only a small variation (8 ‐ 10%) was seen in the measured DO for the slower growing vessels (Figure 5 ii).

Irrespective of the difference in growth rate during exponential phase of the cells in CS2, the maximum OD obtained was similar to the cells grown in CS1 and Multifors. Product titer from both scales of fermentation was determined at 18 h postinduction (Figure 4B) showing that scFv_1_ exported to the periplasm of the cells was comparable at both scales and cascade control strategies, being 0.42 (cascade B) and 0.65 (cascade A) g L^−1^ for the ambr 15f and 0.55 (cascade B) and 0.61 (cascade A) g L^−1^ for the Multifors system, similar results found at other time points (data not shown); ANOVA test showed that at a level of 0.05 there was no significant difference in means of product titer by control strategy used or scale (1 L and 15 mL). In addition, the product in the extracellular medium at both scales was less than 0.05 g L^−1^.

Figure 5 shows the traces from the control cascade A and B strategies used in both the Multifors and ambr 15f. As expected, there were significant differences in the P/V (Figure 5 iii and iv), air (Figure 5 v and vi) and oxygen gas flow rates (Figure 5 vii and viii) traces between the two control cascades. For P/V, the coefficient of variance within the 12 vessels in each CS and between the two CS was below 1.5% (Figure 5 iii and iv). In the case of air gas flow rate, the coefficient of variance was 8% (Figure 5 v) and 14% for cascade A and cascade B respectively (Figure 5 vi), demonstrating that in this scenario, control cascade A lead to less variability.

Figure 5 shows that there is a great deal of similarity between the two bioreactor formats for cascade control strategy A. It is worth noting that a simplification in this control approach meant that once the maximum stirrer speed was reached (Figure 5 iii), this value was held, therefore while the P/V increases over time during the exponential growth phase of the culture, the P/V subsequently decreases for the Multifors system, as air flow rate was enough to maintain DO set point, whilst it remains at this maximum level for the ambr 15f system. It is also worth noting that the higher observed k_L_a in the ambr 15f system is observed where at matched peak P/V, a greater proportion of oxygen flow is required in the Multifors system as compared to the ambr 15f bioreactor vessel (Figure 5 vii).

There is less similarity between the plots for cascade control strategy B whilst the comparison looks good toward the beginning of the fermentation; this breaks down after 15 h. At this stage an imbalance in the control strategy occurs in the ambr 15f system leading to excess oxygenation (Figure 5 ii), likely to be the cause of reduced growth rate observed in Figure 4A. It is possible that further tuning of the PID terms used in the process parameters may help to alleviate this issue however this was not observed in this study.

The pH showed similar tends and ranges, during batch phase of the fermentation (20 h for cascade A and 26 h for cascade B). The pH of the ambr 15f system (Figure 5 ix and x) was controlled within the range of the Multifors and equal percentage base was added at both scales (Figure 5 xi and xii). However, in the fed‐batch phase there was an increase in the % of base (v/v) added to the ambr 15f.

There was no significant difference in the total evaporation volume losses for cascade A and B. At the end of fermentation the total evaporation for vessels in either cascade A and B was 5% of the total volume. As the evaporation rates during this experiments were minimal, and given the intrinsic variability of the analytical methods (i.e., Optical density measurements), it was decided that there was need to compensate for evaporation. Evaporation losses will have a more significant effect at smaller scale which can have knock‐on effects on osmolality, mass transfer, for example. In turn, this can affect cell growth and metabolism.

In summary, we showed that the ambr 15f does support comparable culture performance in terms of cell growth, maximum optical density and productivity as the 1 L Multifors system. From the two cascade strategies tested to control dissolve oxygen we demonstrated that cascade A, a more traditional cascade system, was more suitable to control ambr15 f system.

### Adaptation of fed‐batch feeding strategy for use with alternative carbon sources

**Figure 6:**
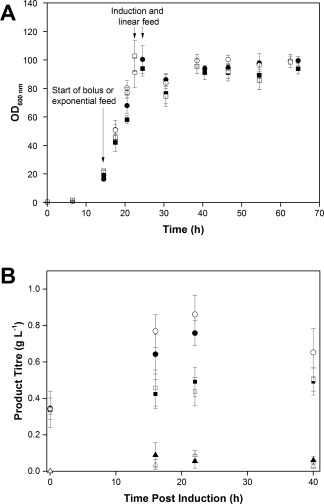
Comparison of feeding strategies and carbon source on *E. coli* MC4100 cells grown fed‐batch fermentation in ambr 15f system. **A**: Cells were fed with 40% (w/w) glycerol (▪,□) or 40% (w/w) glucose (○,•) using bolus additions (empty symbols) or exponential feeding (filled symbols) until they reach an OD of 100, then constant feeding (3.2 mL L^‐1^ h^‐1^) was used. *n* = 6 for each condition tested. **B**: scFv_1_ concentration in the extracellular medium (M), periplasmic (P) and cytoplasm (C) fractions determined by HiTrap protein L HPLC. Vessels quantified/analyzed *n* = 6 for 16 and 40 h postinduction and *n* = 2 for 0 and 22 h postinduction.

The supplementation of glucose or glycerol was performed as either bolus additions or exponential feeding. We tested the how this automated system could implement the use of alternative carbon sources (e.g., replacing glycerol with glucose). Glucose concentration in the medium was limited to 30 g L^−1^ to reduce the accumulation of acetate and its inhibitory effects on cell growth and productivity.^39^, ^40^ To keep the same conditions, the concentration of glycerol in the medium was also reduced to 30 g L^−1^. The two carbon sources were separately tested in each culture station (Figure 1A); when the carbon source was exhausted (indicated by pH spike) the individual vessel was supplemented with its respective carbon source and feeding strategy to an equivalent total concentration of 90 g L^−1^. Figure 6A shows that both feeding strategies tested were suitable to achieve similar high OD values. As expected,^41^ the cells grown in medium with glucose had a slightly higher maximum growth rate compared to the ones grown in glycerol medium (Table 2), ANOVA test showed that at a level of 0.05 there was significant difference in the population means of growth rate, by the carbon source or feeding strategy. Cells cultured using a bolus feeding strategy, irrespective of the carbon source, reached the stationary phase faster (22 h post inoculation) while the exponential fed cells reached stationary phase 24 h post inoculation. This shows that the feeding strategy has an important effect on the cell metabolism. In this case, the exponential feeding strategy limits the amount of glucose/glycerol available at a specific time in culture, thereby slightly reducing the growth rate for either carbon source. In all the conditions tested, the maximum OD_600 nm_ was approximately 100 (Figure 6A), which was maintained for 40 h. The DCW at harvest was comparable for the glucose and glycerol fed cells (Table 2). This performance was similar to the one shown by the cells grown in medium with initial concentration of glycerol of 90 g L^−1^ (Figure 2A) proving that the ambr 15f system was suitable for comparison of medium composition and feeding strategy. We showed that the ambr 15f can be used to implement automated feeding, which will allow better feeding control and benefit cultures were metabolites or by products inhibit growth.^22^, ^23^

**Table 2:** Fermentation Parameters from *E. coli* MC4100 Expressing a scFv_1_ Grown in Minimal Medium with Glucose or Glycerol as Carbon Source

					Periplasmic product (g L^−1^)		
Carbon source	Feeding during exponential phase	Fermentation time (h)	Induction time (h)	*μ*_max_ (h^−1^)	16 h PI	40 h PI	DCW at harvest (g L^−1^)
Glucose	Exponential	64.5	24	0.29 ± 0.005	0.76 ± 0.10	0.76 ± 0.06	33.5 ± 0.7
Glucose	Bolus	62.5	22	0.30 ± 0.004	0.78 ± 0.09	0.73 ± 0.29	31.5 ± 2.0
Glycerol	Exponential	64.5	24	0.26 ± 0.005	0.62 ± 0.12	0.69 ± 0.09	34.1 ± 2.1
Glycerol	Bolus	62.5	22	0.27 ± 0.001	0.67 ± 0.10	0.61 ± 0.16	35.5 ± 2.3

PI: Time postinduction, *n* = 6.

The volume availability in the ambr 15f microbioreactor allowed time course samples to be taken to determine the cells productivity over the fermentation. Figure 6B shows that there was a similar productivity for cells grown in glucose or glycerol medium. At induction, protein concentration in the periplasm and cytoplasm was similar, however later in culture there was a higher concentration of scFv_1_ in the periplasm than in the cytoplasm; ANOVA test showed that there was not a significant difference (at a level of 0.05) in the mean product titer by the cells fed with glucose or glycerol. The maximum product concentration in the periplasm was achieved 22 h postinduction, indicating that the harvest time could be optimized at the small scale. In addition, little product was found in the extracellular medium, confirming data found in the shear studies, that cells in the small scale system were resistant to damage.

### Strain and product screening

**Figure 7:**
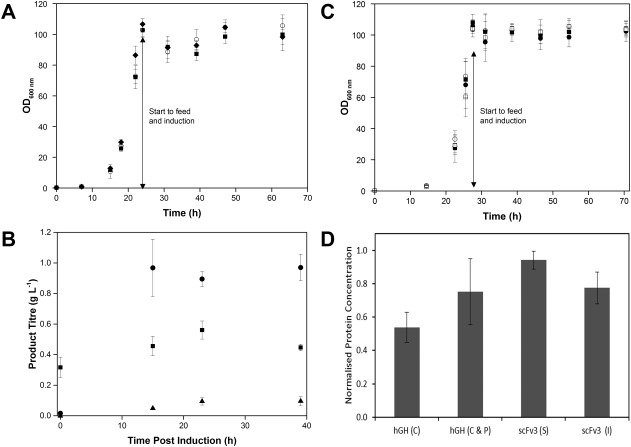
Screening of alternative strains and molecules in ambr 15f system. **A**: Growth curves during fedbatch fermentations of *E. coli* MC4100 cells expressing scFv_1_ (○), scFv_2_ (▪) and scFv_2_* (filled diamonds) (*n* = 8). **B**: scFv_1_ concentration in the periplasm (●), cytoplasm (▪) and extracellular medium (filled triangles) determined by protein L HPLC at 0, 15, 23, and 39 h post induction at harvest (vessels quantified/analyzed: *n* = 2 for 0 and 23 h and *n* = 8 for 15 and 39 h). **C**: Growth curves during fed‐batch fermentations of *E. coli* BL21 (DE3) cells expressing scFv_3_ (S) (▪) scFv_3_ (I) (□) and hGH (C) (●) hGH (I & P) (○), *n* = 6 for each molecule. **D**: Normalised protein concentration at harvest (42 h postinduction) determined by IMAC HPLC, vessels quantified/analyzed: *n* = 6 for each molecule.

*E. coli* MC4100 cells expressing different scFv molecules (scFv_1_, scFv_2_, scFv_2*_) and *E. coli* BL21 (DE3) cells expressing hGH and scFv_3_ were tested in the ambr 15f. The evaluation of the molecules was performed in replicates of 8, with 4 vessels in each CS for MC4100 cells, and in replicates of 6, with 3 vessels in each CS for BL21 (DE3) cells. The positions of the molecules was randomized in the ambr 15f but kept the same in the both culture stations while all the fermentation parameters were maintained the same for all vessels. There was comparable growth for the MC4100 cells expressing different types of scFv (Figure 7A) reaching a maximum OD_600 nm_ of 100 that was maintained until harvest. The *μ*_max_ was 0.26 ± 0.006 h^−1^ (At a level of 0.05 t test showed that there was no significant difference from the population mean) and DCW at harvest 37.6 ± 2.3 g L^−1^. However, the expression of the different products was dissimilar; densitometry analysis showed that there was good expression of scFv_1_ (∼0.64 g L^−1^), low expression of scFv_2_ (∼0.07 g L^−1^) and no bands were detected for scFv_2*_. Further quantification of expression of scFv_1_ was performed by Protein L HPLC (Figure 7B); indicating a similar concentration in the periplasm and cytoplasm at induction and the product was efficiently exported to the periplasm of the cells during the fermentation with little leakage into the extracellular medium. Figure 7C shows that the growth of BL21 (DE3) cells expressing different molecules was comparable (*μ*_max_ 0.22 ± 0.004 h^−1^, with no significant difference from the mean at level of 0.05, DCW at harvest 37.8 ± 1.7 g L^−1^). The total protein concentration at harvest was determined by IMAC HPLC (Figure 7D) showing that there was a comparable product expression. This demonstrates that the ambr 15f system clearly resolves between protein expression performance of different strains and can be used as an effective high throughput tool for screening of molecules and strains.

## Conclusion

We have shown that the microbial ambr 15f system is capable of supporting microbial fed‐batch fermentations to high cell concentrations similar to benchtop and pilot scale fermenters. The growth and productivity profile of all 24 microbioreactors were comparable within and across culture stations. We demonstrated that a pH spike can be used as a signal to activate automated system functions; in this case, a switch in the DOT control loop, triggering actions of the pumped liquid delivery system (i.e., feeding) or the liquid handler (sampling and induction). In addition, we developed fed‐batch feeding strategies (bolus and exponential) that allowed the use of glucose as an alternative carbon source to glycerol. We showed that the ambr 15f system mimics the growth and productivity of the 1 L fermenters (which was previously shown to be scalable to 20 L) and that the cells grown in the ambr 15f system are as resistant to damage as those in larger fermenters. Additionally, we showed that a more conventional cascade control strategy, i.e., cascade A (air/stirrer/oxygen) would be recommended for better control of the ambr 15f system and optimum cell growth. In summary, the ambr 15f was used successfully as a high throughput fermentation system for screening of molecules and microbial strains and as a scale down system that proved to be a reproducible, controllable way of growing and expressing industrially relevant molecules. We have shown that the ambr 15f is a robust, consistent high throughput system, which provides a solid base to carry out DoE. This would ultimate lead to a great advantage in process/product development as time lines would be shortened. As in any high throughput multi‐vessel system useful for upstream development, the limitation resides in the time needed to process the samples for analytical analysis. To create a truly high speed integrated process development, the need remains to match the upstream with similar high throughput downstream process operations.

## Supporting information

Additional Supporting Information may be found in the online version of this article at the publisher's website.

Supporting Information Table 1.
