# Supplementary material for: High throughput automated microbial bioreactor system used for clone selection and rapid scale‐down process optimization
Source: Biotechnol Prog. 2017 Aug 10;34(1):58–68. doi: 10.1002/btpr.2534 (PMC5836883; doi:10.1002/btpr.2534)
Supplement: Supplementary file 1 — Supporting Information Table 1. [file BTPR-34-58-s001.docx]

Appendix A

Table S1. Abridged k_L_a (h^-1^) characterisation for the ambr 15f (data courtesy of Sartorius Stedim Biotech)

| Fill volume (mL) | Agitation rate (rpm) | Gassing at  7 mL min^-1^ | Gassing at  18.5 mL min^-1^ |
| --- | --- | --- | --- |
| 7 | 1000 | 23 |  |
|  | 2000 |  | 210 |
|  | 3000 | 53.4 |  |
| 11 | 1000 |  | 20 |
|  | 2000 | 150 | 180 |
|  | 3000 |  | 180 |
